# Physics-informed latent-space optimization for energy-aligned hole-collecting monolayers in inverted perovskite solar cells

**DOI:** 10.1039/d6ra03728c

**Published:** 2026-08-06

**Authors:** Naomu Sekiguchi, Satoshi Iikubo

**Affiliations:** a Department of Advanced Materials Science and Engineering, Faculty of Engineering Sciences, Kyushu University 6-1 Kasuga Koen, Kasuga Fukuoka 816-8580 Japan iikubo.satoshi.472@m.kyushu-u.ac.jp +81-92-583-7943

## Abstract

Precise energy-level alignment at buried interfaces is critical for high-performance inverted perovskite solar cells, where the highest occupied molecular orbital (HOMO) of hole-collecting monolayers (HCMs) must be tuned relative to the valence band maximum (VBM) to enable efficient hole extraction while minimizing energy loss. Systematic exploration of suitable molecular structures remains challenging because of the vast chemical design space and the computational cost of first-principles screening. A physics-informed generative molecular design framework is developed in which HCM discovery is formulated as an energy-alignment–constrained optimization problem. A high-accuracy HOMO prediction model trained on a large-scale first-principles dataset achieves a mean absolute error of 88 meV and demonstrates transferability to previously reported HCM molecules. The predictor is integrated with a variational autoencoder (VAE) to construct a continuous molecular latent space, where Bayesian optimization is performed using an objective function that explicitly encodes HOMO–VBM alignment, structural similarity to known HCM motifs, and anchoring-group requirements. Latent-space optimization selectively identifies candidate molecules that satisfy energy-level alignment while retaining key structural features of known HCMs and extending beyond existing chemical space. The resulting candidates exhibit both structural novelty and functional relevance, demonstrating that the framework enables directed and physics-guided exploration of molecular design space. This approach demonstrates the potential of combining molecular generation, HOMO prediction, and Bayesian optimization for HCM candidate discovery in inverted perovskite solar cells.

## Abstract 2

Precise energy-level alignment at buried interfaces is critical for efficient inverted perovskite solar cells, requiring the highest occupied molecular orbital (HOMO) of hole-collecting monolayers (HCMs) to align with the valence band maximum (VBM).

## Graphical abstract

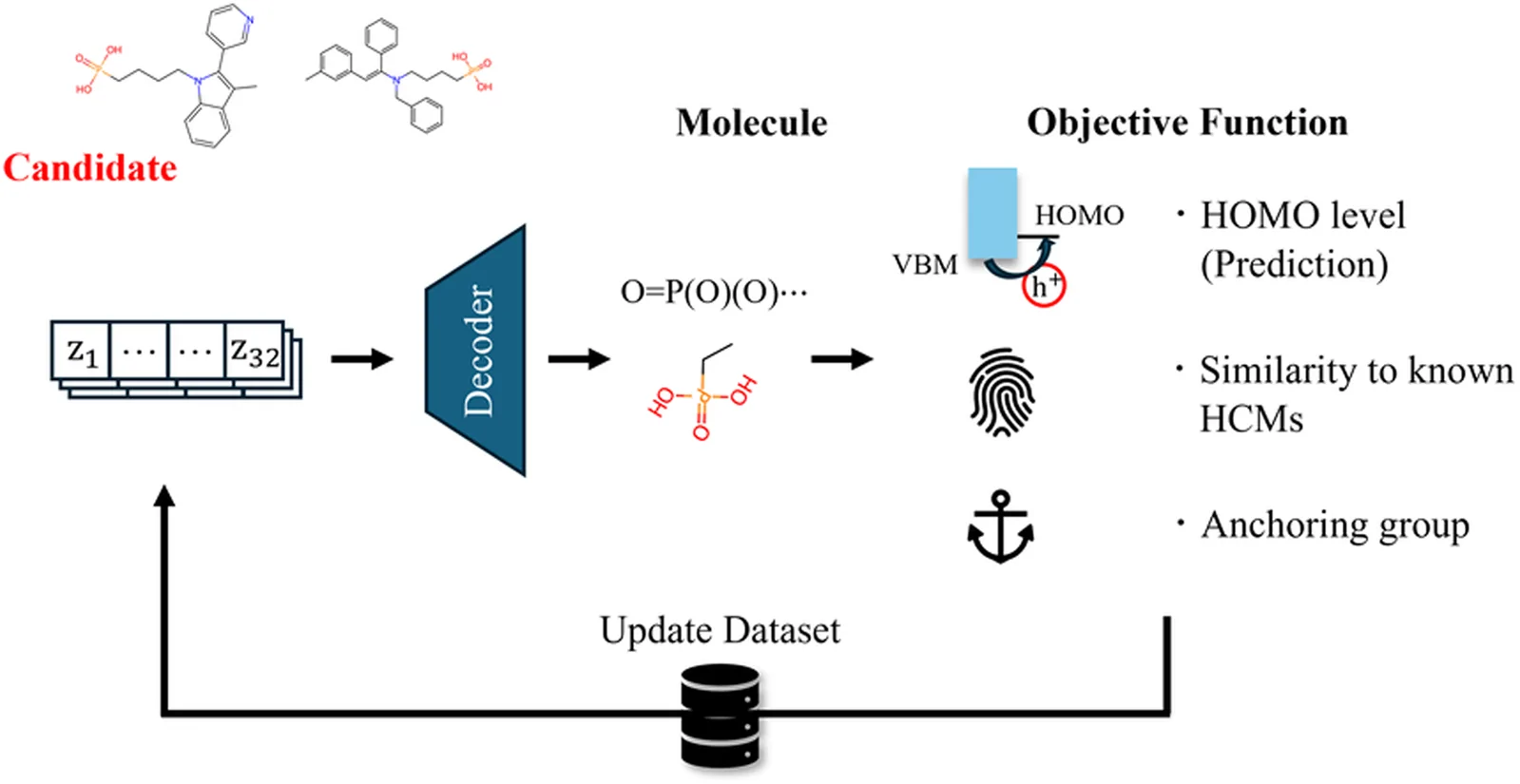

## Introduction

Perovskite solar cells (PSCs) have rapidly emerged as a leading photovoltaic technology, achieving certified power conversion efficiencies exceeding 26% ^1^ while maintaining remarkable tunability in composition and device architecture. In particular, inverted (p–i–n) architectures employing self-assembled hole-collecting monolayers (HCMs) have attracted increasing attention due to their reduced parasitic absorption, improved interfacial stability, and compatibility with tandem device integration.^2–22^ Unlike conventional polymeric hole transport layers such as PEDOT:PSS,^23,24^ HCMs form ultrathin molecular monolayers directly anchored to the substrate, enabling precise control of interfacial energetics and charge extraction.

Among the various factors governing HCM performance, the energetic alignment between the highest occupied molecular orbital (HOMO) of the HCM and the valence band maximum (VBM) of the perovskite absorber plays a central role. Improper alignment can lead to interfacial charge accumulation, increased recombination, and voltage losses.^25^ Experimental and theoretical studies have shown that excessively deep HOMO levels relative to the perovskite VBM may hinder efficient hole extraction, whereas overly shallow levels can compromise interfacial selectivity. Therefore, rational design of HCM molecules requires precise tuning of their HOMO energy to achieve optimal alignment with the perovskite electronic structure.

Traditionally, such energy-level optimization relies on density functional theory (DFT) calculations. While DFT provides reliable electronic structure predictions, high-throughput screening of large molecular libraries remains computationally prohibitive, particularly for relatively large π-conjugated molecules typically used in HCMs. Consequently, molecular design has largely relied on chemical intuition and incremental structural modifications of known scaffolds, limiting exploration of broader chemical space. Recent advances in deep learning have enabled data-driven prediction of molecular properties and generative design of novel compounds.^26^ Variational autoencoders (VAEs) and related generative models allow construction of continuous latent spaces in which new molecular candidates can be sampled and optimized. However, many existing generative approaches focus primarily on statistical property optimization without explicitly incorporating physically meaningful design constraints derived from device physics. As a result, the generated molecules may not satisfy the stringent energetic requirements necessary for interfacial charge transport in PSCs.

In this work, we develop a physics-informed generative design framework for discovering novel HCM candidates with optimized energy-level alignment for inverted perovskite solar cells. First, we construct a high-accuracy HOMO prediction model trained on a large-scale first-principles dataset of organic molecules.^27,28^ The model is designed to accommodate relatively large molecular structures relevant to HCM applications. Second, we build a VAE-based molecular generative model that maps organic molecules into a continuous latent space, enabling systematic exploration beyond known compounds.^26^ Third, we integrate these components through Bayesian optimization in the latent space, where the objective function explicitly incorporates (i) HOMO–VBM alignment, (ii) structural similarity to known HCM motifs,^2–22^ and (iii) the presence of anchoring groups necessary for substrate binding.

By embedding physically motivated design criteria directly into the generative optimization process, our approach enables targeted exploration of molecular space guided by device-relevant energetic principles rather than empirical heuristics. This framework provides not only new HCM candidates but also insights into the structural factors governing energy-level alignment, thereby establishing a general strategy for physics-guided molecular discovery in perovskite optoelectronics.

## Computational methods

For the supervised learning of HOMO levels, we utilized a dataset computed by M. Nakata *et al.*,^27,28^ in which HOMO levels of organic molecules were calculated using DFT (B3LYP/6-31G*) based on entries in the PubChem database. The training set consisted of 1.9 million organic molecules, with 60 000 and 40 000 molecules allocated to the validation and test sets, respectively. A chemical language model was adopted to represent molecular structures as one-dimensional character strings for input, using SMILES2vec.^29^ Specifically, we employed the Atom-in-SMILES (AIS) format,^30^ which has been reported to outperform other models in terms of property prediction accuracy and minimizes information loss during the string conversion process. To ensure uniqueness of molecular representations, we used canonical SMILES generated by RDKit. For the HOMO level prediction model, we followed a deep learning architecture similar to that proposed by R. J. Richards *et al.*^31^

**Fig. 1:**
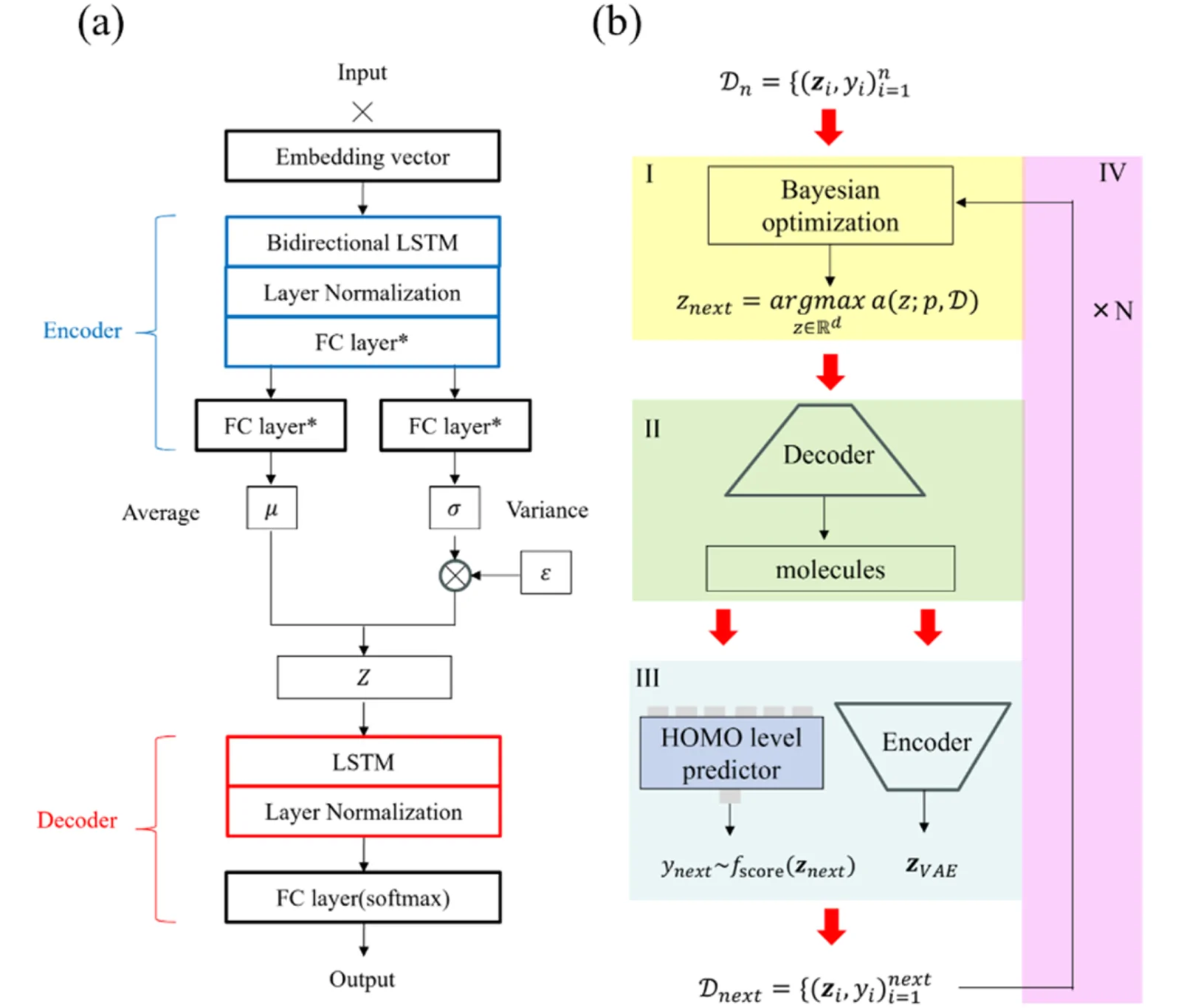
(a) Architecture of the deep learning model for the variational autoencoder (VAE). (b) Workflow of candidate HCM material discovery using Bayesian optimization.

The VAE model also used string-based inputs and outputs in the AIS format, and the deep learning architecture was based on an RNN model design inspired by the work of O. Dollar *et al.*^32^ The training data for the VAE consisted of 1.9 million molecular structures randomly selected from the PubChem database, with a restriction that the number of AIS tokens be 200 or fewer. This constraint was imposed to match the input length used in the HOMO prediction model. The latent space dimensionality of the VAE was set to 32, considering the computational cost of Bayesian optimization performed in the latent space. The validation and test sets for the VAE model were the same size as those used in the HOMO prediction model, and reconstruction accuracy and validity were evaluated at each epoch. As shown in Fig. 1(a), the VAE architecture incorporated LSTM layers. For both models, model weights were optimized using the Adam optimizer.^33^

To guide molecular exploration toward physically relevant HCM candidates, we defined an objective function that explicitly incorporates energy-level alignment, structural relevance, and anchoring capability. For a molecule generated from a latent vector z, the objective function 
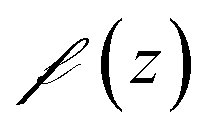
 is defined as:1
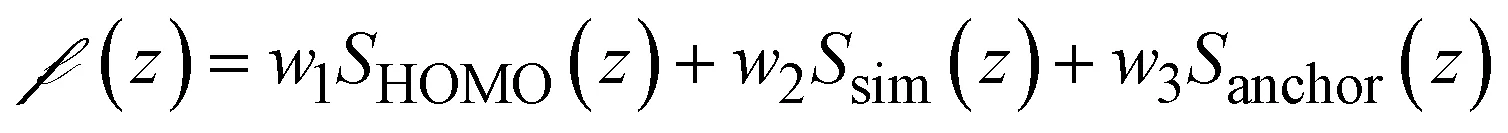
where *S*_HOMO_, *S*_sim_, and *S*_anchor_ represent the HOMO alignment score, structural similarity score, and anchoring-group score, respectively. In this study, all terms were normalized to the range [0,1], and equal weights were adopted (*w*_1_ = *w*_2_ = *w*_3_ = 1) as a simple baseline scoring scheme. We note that the three score components have different statistical characteristics: *S*_HOMO_ is a continuous Gaussian score, *S*_sim_ is a continuous similarity score, and *S*_anchor_ is a binary descriptor. Therefore, the anchoring-group term may have a relatively large influence on the objective function. Nevertheless, anchoring groups are essential for HCM/SAM molecules because they enable stable adsorption on electrode surfaces and contribute to the formation of hole-selective interfacial layers. Thus, the anchoring-group term was intentionally included in the objective function for primary screening of HCM candidates.

The HOMO alignment score was defined using a Gaussian function centered at a reference energy *E*_ref_:2
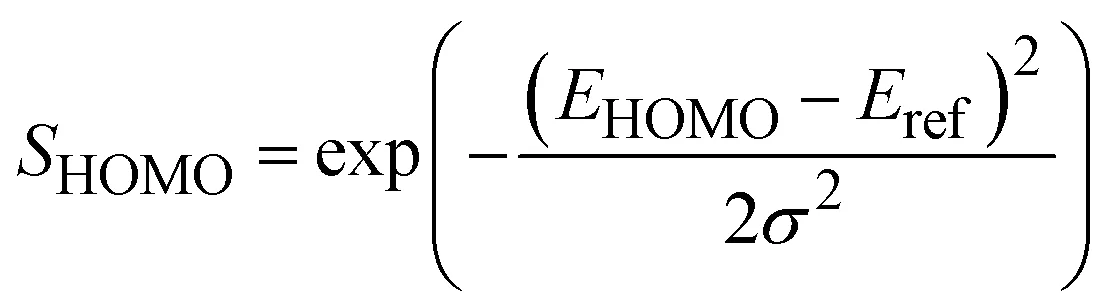
where *E*_HOMO_ is the HOMO energy predicted by the trained model, *E*_ref_ = −5.4 eV, and *σ* = 0.25 eV.

The reference energy was selected considering that the valence band maximum (VBM) of typical perovskite absorbers lies around −5.6 to −5.5 eV relative to vacuum. A HOMO level slightly shallower than the VBM is generally favorable for efficient hole extraction while minimizing interfacial energy loss.^34,35^ The Gaussian form provides a smooth and differentiable scoring landscape, enabling stable optimization in latent space while penalizing energetically mismatched candidates.

To maintain chemical relevance to known high-performance HCM motifs, a structural similarity score was introduced:3
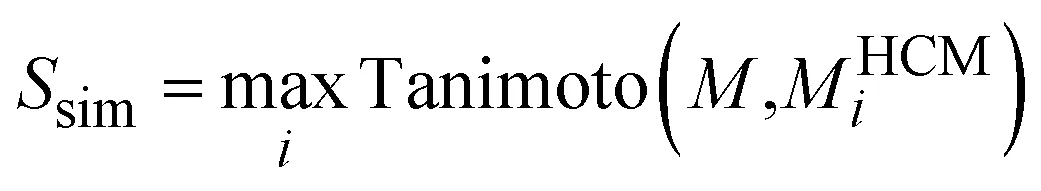
where *M* is the generated molecule and *M*^HCM^_*i*_ denotes previously reported HCM structures. The Tanimoto similarity^36^ was computed using Morgan fingerprints^37^ with radius *r* = 2. The maximum similarity value across all reference HCM molecules was adopted. This term prevents the optimization from drifting toward chemically unrealistic regions of latent space while still allowing structural innovation beyond existing compounds.

Since effective HCM molecules require anchoring groups to bind to the substrate surface, phosphonic acid and carboxylic acid groups were regarded as valid anchoring groups. Accordingly, we defined:4
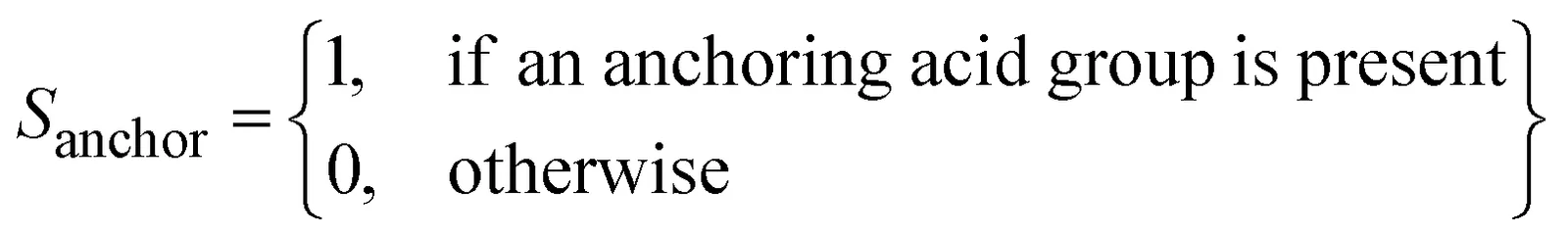


If the generated structure was chemically invalid or could not be decoded into a valid molecule, the objective function was assigned a penalty value of −10, ensuring that invalid candidates were excluded from further optimization.

The objective function was constructed to encode device-relevant physical requirements directly into the molecular optimization process. Rather than optimizing a purely statistical descriptor, the search is guided by (i) physically motivated energy-level alignment, (ii) structural compatibility with known HCM motifs, and (iii) essential anchoring functionality. By integrating these constraints into Bayesian optimization in the VAE latent space, the framework enables targeted exploration of chemically meaningful and energetically suitable HCM candidates.

Next, the Bayesian optimization procedure used for molecule exploration is described. As illustrated in Fig. 1(b), the process follows a stepwise flow:

I. Bayesian optimization is used to identify the latent vector that maximizes the acquisition function, based on the objective function and the latent space.

II. The obtained latent vector is then passed through the decoder of the pretrained variational autoencoder (VAE) to generate a corresponding molecular structure.

III. The dataset is updated with the newly generated molecule, its associated objective function value, and the corresponding latent vector (calculated from the mean without standard deviation).

IV. Bayesian optimization is performed again using the updated dataset.

By repeating this iterative process, it becomes possible to efficiently explore high-performance candidate HCM materials with high objective function values. The initial set of molecular samples is selected randomly and incorporated into the process using the same procedure described above. In this study, the acquisition function used for optimization was the Upper Confidence Bound (UCB), and its hyperparameter was set to *β*_*n*_ = 0.5 to balance exploration and exploitation during the search.

## Results and discussion

**Fig. 2:**
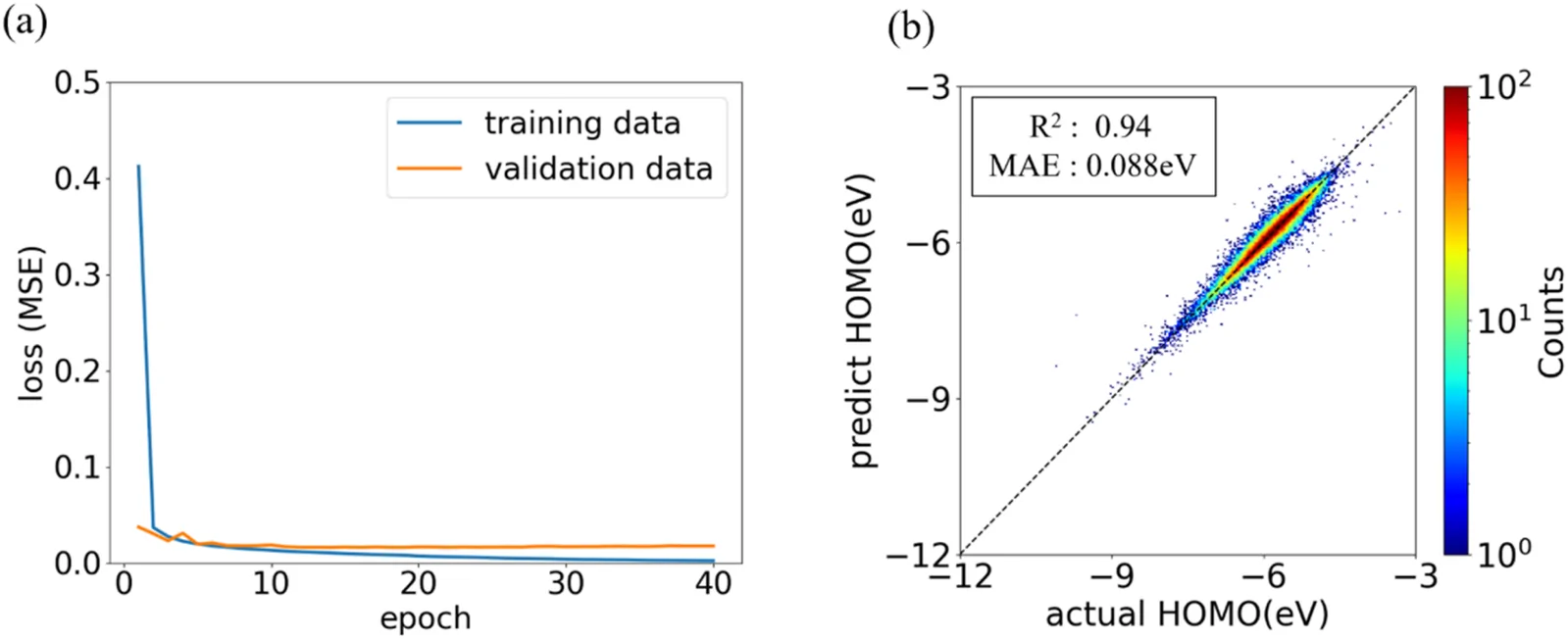
(a) Loss values for the training and validation datasets at each epoch during HOMO level prediction. (b) Prediction results for HOMO levels on the test dataset.

**Fig. 3:**
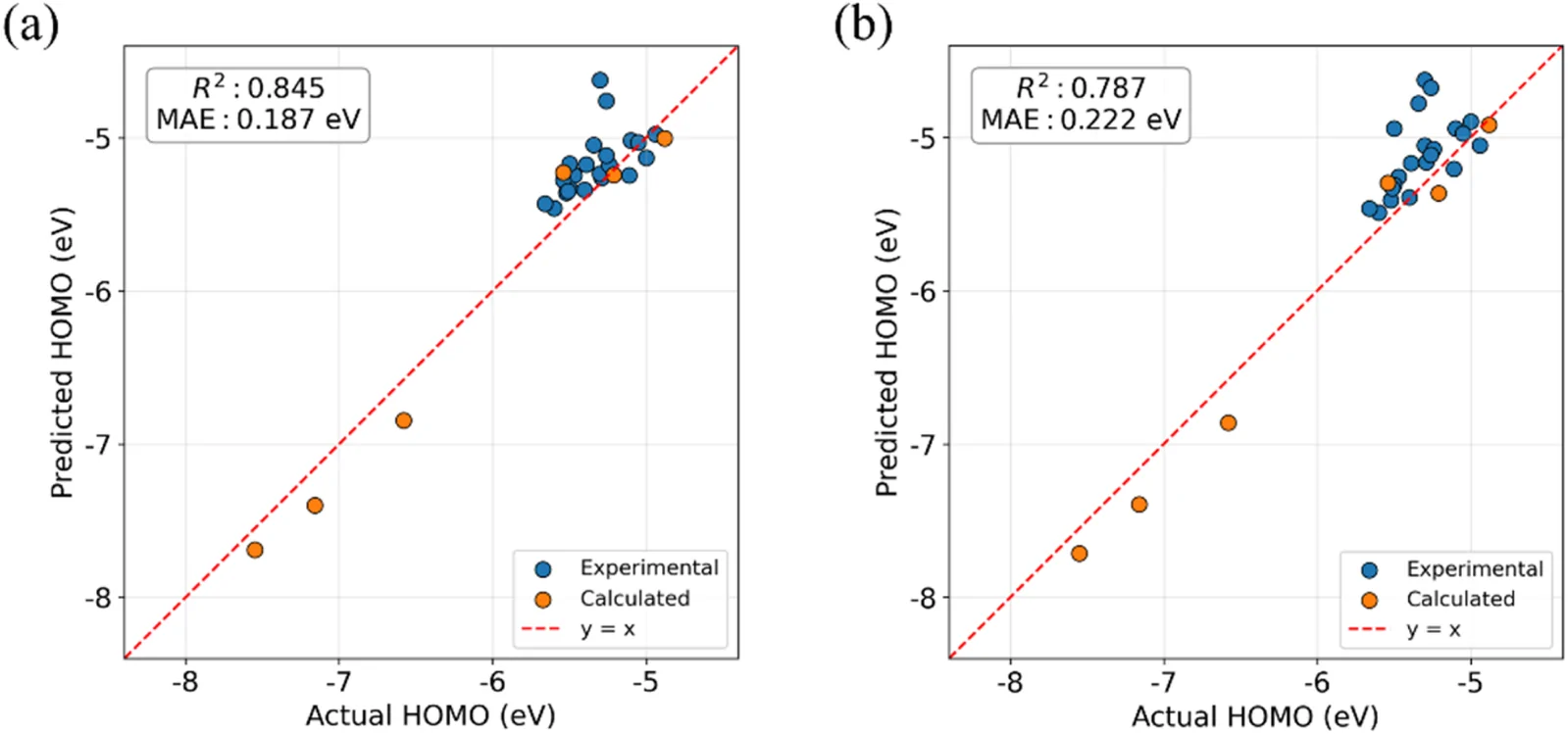
(a) Predicted HOMO levels of HCMs using a chemical language model. (b) Predicted HOMO levels of HCMs using a graph model.

For HOMO level prediction, the initial learning rate was set to 1.0 × 10^−3^, and a learning rate decay strategy was employed: the rate was halved if the mean absolute error (MAE) on the validation dataset did not improve for five consecutive epochs. As shown in Fig. 2(a), the model training was terminated at epoch 40, as no further improvement was observed in the validation loss. At this point, the model achieved a MAE of 88 meV and a coefficient of determination (*R*^2^) of 0.94 on the test dataset, indicating high predictive accuracy (Fig. 2(b)). Although this accuracy is lower than that of graph neural network (GNN) models such as SchNet,^38–40^ which use atomic coordinates as input, the SMILES2vec-based model remains highly useful, as it requires only molecular structure information and does not rely on atomic coordinates. On the other hand, a coordinate-free graph model known as the Hierarchical Inter-Message Passing GNN (HIMP-GNN)^41^ demonstrated higher predictive performance, achieving a MAE of 73 meV and *R*^2^ of 0.95—surpassing SMILES2vec in overall accuracy. The reference HOMO levels shown in Fig. 3 were obtained from experimental or computational values reported in the literature for previously studied HCM materials. The experimental methods and computational conditions used to determine the HOMO level of each molecule are summarized in Table S1. Comparison with these reference values confirmed that the chemical language model, SMILES2vec, achieved higher predictive accuracy than the graph-based model, HIMP-GNN. These results indicate that SMILES2vec provides practical predictive accuracy for the HOMO levels of literature-reported HCM molecules. Accordingly, SMILES2vec was adopted as the HOMO-level prediction model in this study.

**Fig. 4:**
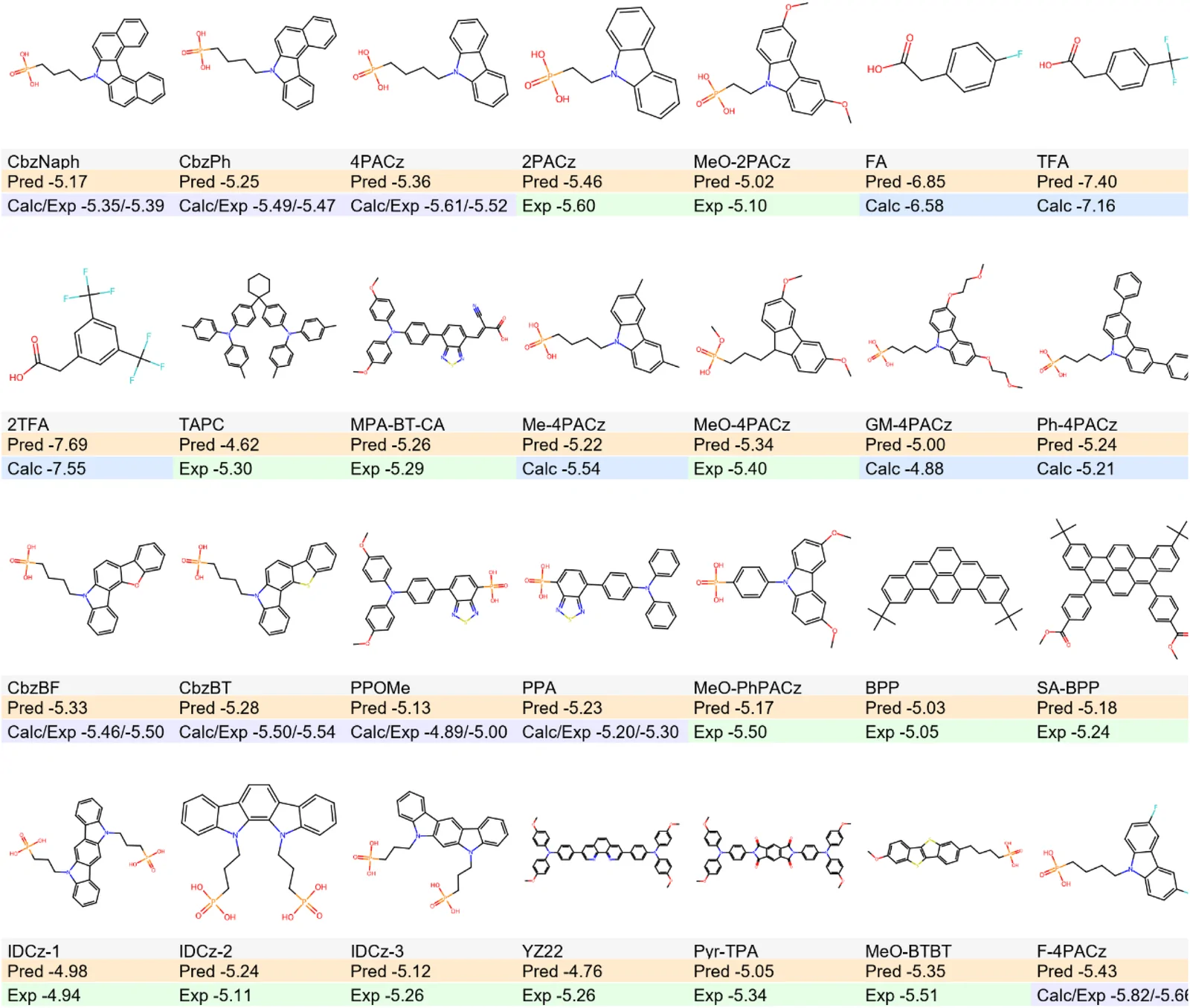
Molecule-by-molecule comparison between HOMO-level predictions by the SMILES2vec model and literature-reported values for representative HCMs. “Pred” denotes the predicted HOMO level, while “Calc” and “Exp” denote calculated and experimentally evaluated reference values, respectively.

To further examine the prediction results for individual HCM molecules, the HOMO levels predicted by SMILES2vec were compared with the corresponding literature-reported values, as shown in Fig. 4. In this figure, “Pred” denotes the HOMO level predicted by the present model, whereas “Calc” and “Exp” represent theoretically calculated and experimentally evaluated HOMO levels reported in the literature, respectively. When both calculated and experimental HOMO levels were available for the same molecule, both values are shown as reference values. The corresponding experimental methods and computational conditions are summarized in Table S1.

Although small quantitative deviations are observed for some molecules, the predicted HOMO levels show reasonable agreement with the reported values. In addition, for the HCM molecules considered in this study, no large systematic deviation is observed between the DFT-calculated HOMO levels and the experimentally evaluated HOMO levels. Therefore, in the present work, we adopted the HOMO levels obtained at the B3LYP/6-31G* level as the training data and did not apply an additional empirical correction to the HOMO levels. These results support the use of SMILES2vec as a practical HOMO-level predictor for guiding subsequent molecular exploration in the VAE latent space. The final reconstruction success rate reached 0.864, with a validity of generated molecules of 0.750. The relatively moderate reconstruction performance is primarily attributed to the low dimensionality of the latent space (32 dimensions), which was intentionally selected to improve the efficiency of exploration in Bayesian optimization. Although higher-dimensional latent spaces can improve reconstruction performance by providing greater representational capacity,^42^ they also expand the search space and increase the cost of Gaussian-process-based optimization, including predictive variance estimation in the UCB acquisition function. Therefore, a 32-dimensional latent space was adopted to maintain sufficient reconstruction performance while enabling practical latent-space exploration.

Using the trained VAE model, 500 000 organic molecules were generated by sampling from a standard normal distribution. The generated molecules exhibited high novelty (0.999) and uniqueness (0.997), indicating that the model is capable of producing structurally diverse molecules beyond the training dataset. Although the validity is lower than that of graph-based generative models,^43–45^ which can achieve near-perfect validity, this is a known limitation of sequence-based molecular representations. Importantly, the generated molecular set provides a sufficiently diverse and novel chemical space for subsequent optimization. Detailed training behavior, including reconstruction success rate, validity trends, and sequence length distributions, is provided in the SI (Fig. S2).

**Fig. 5:**
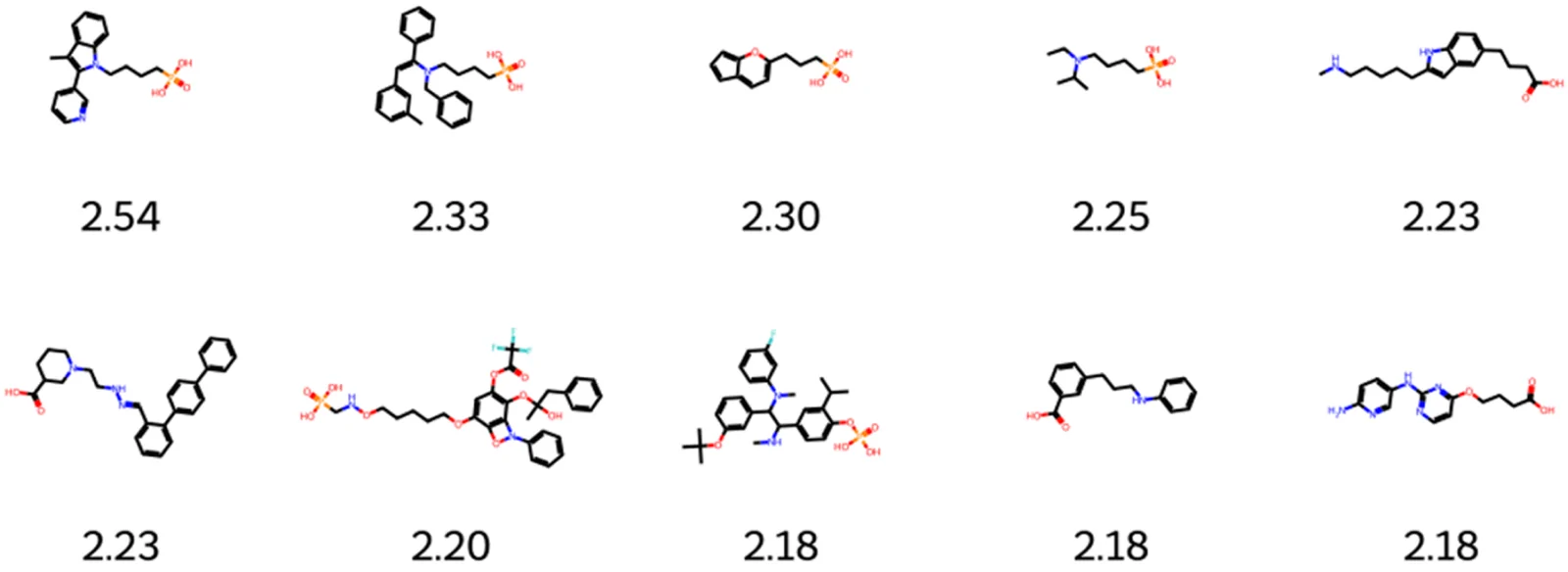
Top-ranked candidate molecules identified by the VAE–Bayesian optimization framework for hole-collecting materials (HCMs). The predicted objective function score are shown below each structure. The candidates exhibit diverse π-conjugated frameworks while retaining structural features commonly observed in known HCMs.

Using the HOMO prediction model and the trained VAE, Bayesian optimization was performed in the 32-dimensional latent space to identify new HCM candidate materials. The objective function was defined to incorporate HOMO–VBM alignment, structural similarity to known HCMs, and the presence of anchoring groups. Using this objective function, Bayesian optimization was carried out for 200 iterations, selecting top candidate points at each step. The optimization progress is summarized in Fig. S3. As shown in Fig. S3(a), the 10-point moving average of the best objective score obtained at each iteration increased as the optimization proceeded, suggesting that the search was gradually guided toward more promising regions in the latent space. A similar trend was observed for the mean objective score in Fig. S3(b), where invalid molecules were assigned a score of zero, indicating that the overall search progressively shifted toward higher-scoring regions. In addition, the validity trend shown in Fig. S3(c) suggests that the optimization increasingly sampled regions capable of generating chemically valid molecular structures. In addition, we performed a post-hoc discovery funnel analysis to clarify the candidate-screening process. Among the 1000 generated candidates, 565 molecules passed the validity check after excluding invalid outputs with a score of −10 or undefined SMILES. All 565 valid candidates were successfully converted into valid molecular representations, and duplicate removal based on canonical SMILES resulted in 564 unique molecules. Among these unique molecules, 233 candidates contained predefined anchoring groups, corresponding to 41.31% of the unique valid molecules and 23.3% of all generated candidates. This filtering process indicates that a substantial fraction of the generated molecules contained chemically relevant anchoring motifs, rather than being arbitrary high-scoring structures. The top-ranked candidate molecules obtained through this process are shown in Fig. 5. These molecules exhibit consistently high objective values, indicating that the optimization successfully identifies promising regions in latent space. Many of the high-scoring molecules contain aromatic or π-conjugated motifs together with polar anchoring groups, structural motifs commonly observed in high-performance HCMs. Several candidates display clear structural resemblance to representative HCM molecules such as 4PACz, while incorporating variations in aromatic substitution patterns and linker geometries. This suggests that the optimization balances structural similarity with molecular diversification, generating modified analogues rather than simple replicas.

To further examine the reliability of the predicted HOMO levels for the generated candidates, geometry optimization and HOMO-level calculations were performed for the top five candidates at the B3LYP/6-31G(d,p) level. The optimized molecular structures are shown in Fig. S4, and the calculated HOMO levels are summarized in Table S2. Among these candidates, molecule 2 showed a DFT-calculated HOMO level that was substantially shallower than those of the other candidates, indicating that its HOMO alignment was overestimated by the prediction model. In contrast, although quantitative differences were observed between the predicted and DFT-calculated HOMO levels for the other candidates, their calculated HOMO levels remained within a suitable range for HCM materials. These results suggest that the proposed optimization framework can identify candidate molecules with potentially favorable HOMO-level alignment, while also highlighting the importance of post-generation DFT validation for final candidate selection.

**Fig. 6:**
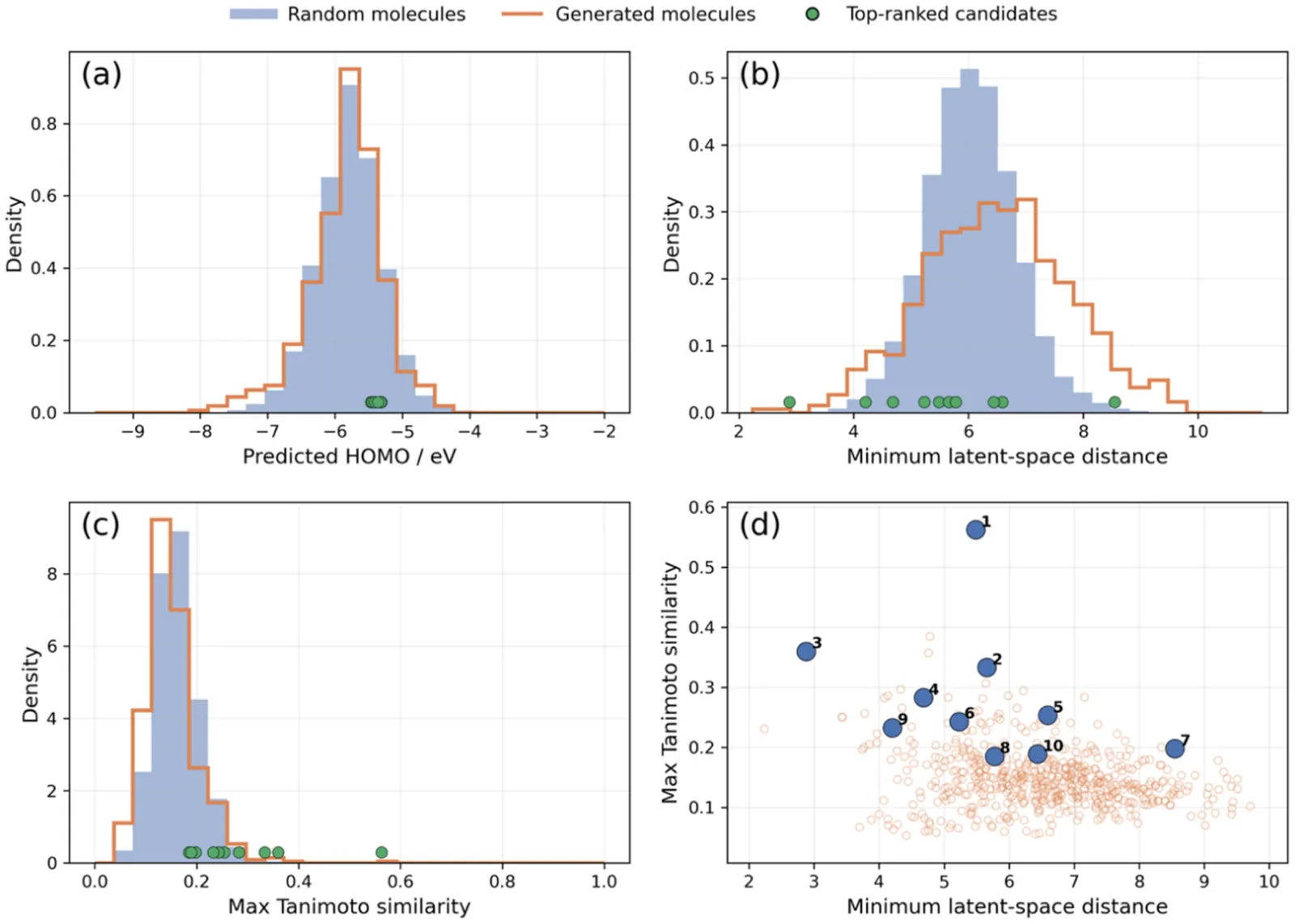
Distributions and relationships characterizing the latent-space optimization of hole-collecting material (HCM) candidates. (a) Distribution of predicted highest occupied molecular orbital (HOMO) energies for randomly sampled molecules, generated molecules, and top-ranked candidates. The overall distribution of generated molecules remains comparable to that of random molecules. (b) Distribution of minimum latent-space distances from reference HCMs, showing that generated molecules occupy broader regions of the latent space. (c) Distribution of maximum Tanimoto similarity to reference HCMs, indicating that most generated molecules are structurally distinct from known HCMs. (d) Relationship between latent-space distance and Tanimoto similarity. While an overall inverse correlation is observed, the top-ranked candidates are systematically located above the bulk distribution, demonstrating that the optimization selectively identifies molecules that retain structural similarity to known HCMs while residing in more distant regions of the latent space.

The distributions of predicted HOMO energies for generated molecules closely resemble those of randomly sampled molecules, indicating that the optimization does not globally bias the electronic property distribution (Fig. 6(a)). Similarly, the distribution of Tanimoto similarity shows that the majority of generated molecules remain structurally distinct from known HCMs (Fig. 6(c)). In contrast, the latent-space distance distribution reveals that generated molecules are systematically located further from known HCMs compared to random samples (Fig. 6(b)), indicating that the VAE–Bayesian optimization framework expands the explored region of chemical space rather than collapsing onto known molecular motifs.

To further elucidate the relationship between structural novelty and similarity, the latent-space distance was plotted against the Tanimoto similarity (Fig. 6(d)). A general inverse relationship is observed, reflecting the consistency between latent-space proximity and structural similarity. Notably, however, the top-ranked candidates are systematically shifted toward higher similarity at comparable distances, indicating that the optimization selectively identifies molecules that retain key structural features of known HCMs while residing in more distant regions of the latent space. These results demonstrate that the proposed framework achieves a balance between structural novelty and functional relevance, enabling the identification of promising HCM candidates beyond the distribution of known molecules. More broadly, this highlights the capability of latent-space optimization to navigate chemical space in a directed and physically informed manner, providing a general strategy for discovering functional molecular materials.

## Conclusion

In this work, we established a physics-informed generative molecular design framework for discovering hole-collecting monolayer (HCM) candidates tailored to inverted perovskite solar cells. By integrating a high-accuracy HOMO prediction model with a variational autoencoder (VAE) and Bayesian optimization in a continuous latent space, molecular discovery was formulated as a constrained energy-alignment problem.

External validation against previously reported HCM molecules confirmed that the HOMO predictor generalizes beyond the training dataset and reliably captures relative energetic trends. Based on this validated predictor, an objective function incorporating HOMO–VBM alignment, structural compatibility with known HCM motifs, and anchoring functionality was constructed. Latent-space optimization using this objective function concentrated candidate molecules within energetically favorable regions while preserving chemically meaningful structures.

Importantly, the framework does not merely retrieve known compounds but generates structurally diverse candidates, including molecules absent from the training dataset. The convergence of top-ranked candidates toward π-conjugated frameworks with appropriate anchoring groups suggests that these features represent essential design elements for energy-aligned HCMs.

Beyond HCM design, this study demonstrates that embedding physically motivated constraints into generative optimization enables directed exploration of chemical space without reliance on empirical heuristics. This approach demonstrates the potential of physics-guided molecular design of interfacial materials in perovskite photovoltaics and related optoelectronic systems.

## Conflicts of interest

There are no conflicts to declare.

## Supplementary Material

RA-016-D6RA03728C-s001

## Data Availability

The code and data supporting this study, including latent representations, SMILES strings, and analysis results, are available at GitHub: https://github.com/Seki706/VAE-and-HOMO-Prediction-Model. Supplementary information (SI) is available. See DOI: https://doi.org/10.1039/d6ra03728c.
