# Supplementary material for: Physics-informed latent-space optimization for energy-aligned hole-collecting monolayers in inverted perovskite solar cells
Source: RSC Adv. 2026 Aug 6;16(40):45964–73. doi: 10.1039/d6ra03728c (PMC13444273; doi:10.1039/d6ra03728c)
Supplement: Supplementary file 1 [file RA-016-D6RA03728C-s001.pdf]

Supplementary Information (SI) for RSC Advances.
This journal is © The Royal Society of Chemistry 2026

Supplementary material

Physics-Informed Latent-Space Optimization for Energy-Aligned Hole-Collecting Monolayers in Inverted Perovskite Solar Cells

Naomu Sekiguchi[1] and Satoshi Iikubo[1,*]

[1]Department of Advanced Materials Science and Engineering, Faculty of Engineering Sciences, Kyushu University, Kasuga, Fukuoka, 816-8580, Japan

Table S1. Summary of reported HOMO energy levels and corresponding computational or experimental conditions for representative HCMs.

Molecule	Reference	HOMO Level	Computational / Experimental Method
CbzNaph	Ref. 2	−5.35 eV / −5.39 eV	DFT (B3LYP/6-311G(d,p)) / Experiment (CV measurement)
CbzPh	Ref. 2	−5.49 eV / −5.47 eV	DFT (B3LYP/6-311G(d,p)) / Experiment (CV measurement)
4PACz	Ref. 2	−5.61 eV / −5.52 eV	DFT (B3LYP/6-311G(d,p)) / Experiment (CV measurement)
2PACz	Ref. 3	−5.60 eV	Experiment (UPS measurement)
MeO-2PACz	Ref. 3	−5.10 eV	Experiment (UPS measurement)
FA	Ref. 4	−6.58 eV	DFT (B3LYP/6-31G level)
TFA	Ref. 4	−7.16 eV	DFT (B3LYP/6-31G level)
2TFA	Ref. 4	−7.55 eV	DFT (B3LYP/6-31G level)
TAPC	Ref. 5	−5.30 eV	Experiment (CV measurement)
MPA-BT-CA	Ref. 6	−5.29 eV	Experiment (CV measurement)
Me-4PACz	Ref. 7	−5.54 eV	DFT (B3LYP/def2-TZVP)
MeO-4PACz	Ref. 8	−5.40 eV	Experiment (UPS measurement)
Br-4PACz	Ref. 8	−5.79 eV	Experiment (UPS measurement)
GM-4PACz	Ref. 9	−4.88 eV	DFT (B3LYP/6-31G(d,p))
Ph-4PACz	Ref. 10	−5.21 eV	DFT (B3LYP/6-31G(d,p))
CbzBF	Ref. 11	−5.46 eV / −5.50 eV	DFT (B3LYP/6-311G(d,p)) / Experiment (CV measurement)
CbzBT	Ref. 11	−5.50 eV / −5.54 eV	DFT (B3LYP/6-311G(d,p)) / Experiment (CV measurement)
PPOMe	Ref. 13	−4.89 eV / −5.0 eV	DFT (PAW-PBE) / Experiment (CV measurement)
PPA	Ref. 13	−5.20 eV / −5.3 eV	DFT (PAW-PBE) / Experiment (CV measurement)
MeO-PhPACz	Ref. 14	−5.50 eV	Experiment (CV measurement)
BPP	Ref. 15	−5.05 eV	Experiment (UPS measurement)
SA-BPP	Ref. 15	−5.24 eV	Experiment (UPS measurement)
IDCz-1	Ref. 18	−4.94 eV	Experiment (CV measurement)
IDCz-2	Ref. 18	−5.11 eV	Experiment (CV measurement)
IDCz-3	Ref. 18	−5.26 eV	Experiment (CV measurement)
YZ22	Ref. 19	−5.26 eV	Experiment (UPS measurement)

Pyr-TPA	Ref. 20	−5.34 eV	Experiment (CV measurement)
MeO-BTBT	Ref. 21	−5.51 eV	Experiment (CV measurement)
F-4PACz	Ref. 22	−5.82 eV / −5.66 eV	DFT (B3PW91/def2-TZVP) / Experiment (CV measurement)

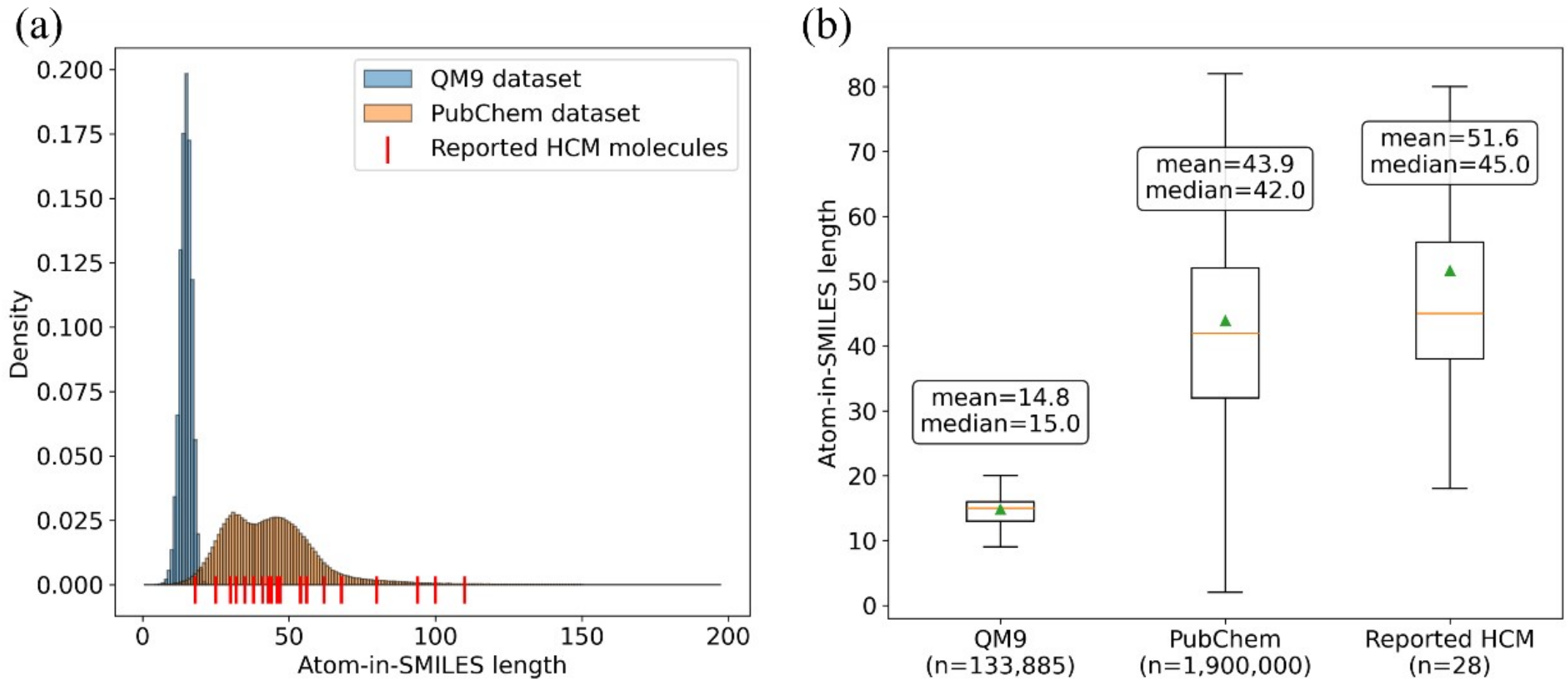

Figure S1. Atom-in-SMILES sequence length distributions. (a) Density histograms for the QM9 reference dataset and the PubChem dataset used for VAE training, with red rug marks indicating reported HCMs listed in Table S1. (b) Box-and-whisker plots comparing the QM9 reference dataset, PubChem VAE training dataset, and reported HCMs. Mean and median values are annotated; outliers are omitted for clarity.

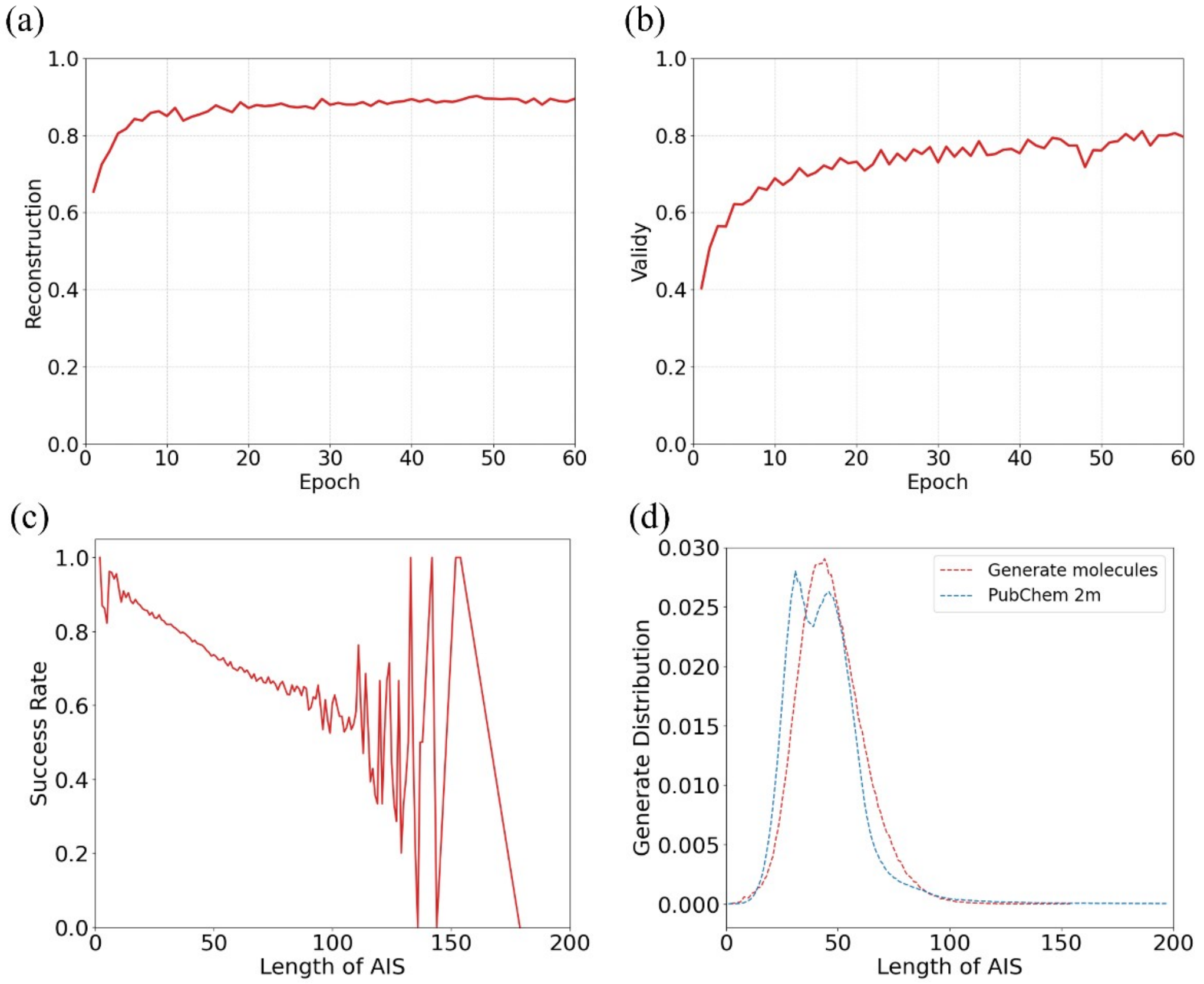

Figure S2. Results of training on 1.9 million organic molecules from the PubChem database. (a) Reconstruction success rate as a function of epoch. (b) Validity probability as a function of epoch. (c) Validity probability as a function of sequence length. (d) Comparison of the token length distributions between generated molecules and the training dataset.

The construction and performance of the VAE model are summarized as follows. The model was trained using an initial learning rate of 1.0×10^{-3}, which was exponentially decayed by a factor of 0.990 at each epoch. The loss function consisted of the Kullback–Leibler (KL) divergence and the reconstruction loss with a weighting factor of $\beta = 0.1$, balancing reconstruction accuracy and latent space regularization.

Bayesian optimization was performed in the 32-dimensional latent space of the trained VAE model. At each iteration, the acquisition function (Upper Confidence Bound) was used to identify latent vectors that maximize the objective function, and the corresponding molecular structures were obtained by decoding the latent representations. At each iteration, the top candidate points were selected based on the acquisition function, and the optimization was repeated for a total of 200 iterations.

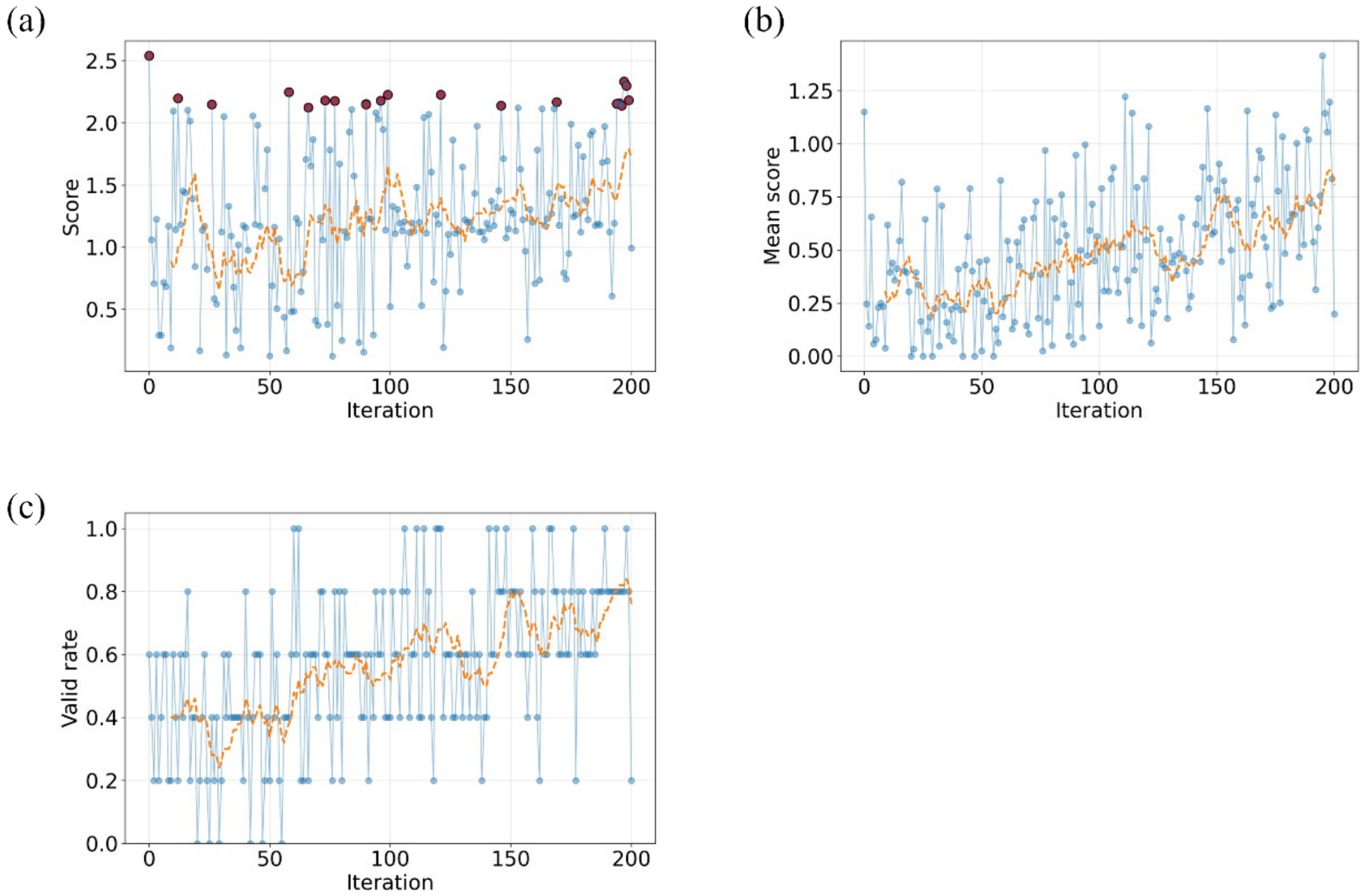

Figure S3. Evolution of molecular optimization during the VAE–Bayesian optimization process. (a) Best objective score at each iteration together with the 10-iteration moving average. Red circles denote the top 20 candidate molecules. (b) Mean objective score at each iteration and its 10-iteration moving average, calculated by assigning a score of zero to invalid candidates. (c) Validity of generated molecules at each iteration and its 10-iteration moving average.

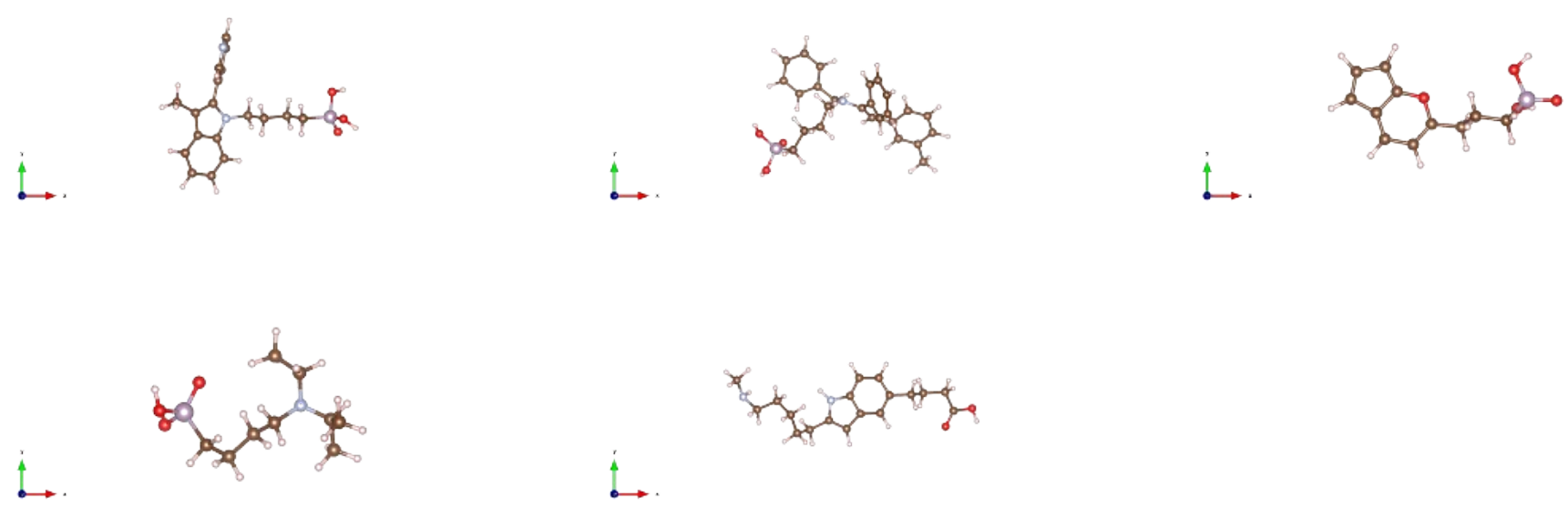

Figure S4. Optimized molecular structures of the top five HCM candidates with the highest acquisition function values.

Table S2. HOMO levels of the top five HCM candidates with the highest acquisition function values.

top	Predict	B3LYP/(d, p)
1	-5.35eV	-5.33eV
2	-5.41eV	-4.86eV
3	-5.31eV	-5.10eV

4	-5.47eV	-5.20eV
5	-5.34eV	-5.22eV

The molecular geometries were optimized at the B3LYP/6-31G(d,p) level of theory, and the HOMO levels were calculated using the optimized structures.
